# Multidisciplinary team’s experiences caring for older adults in the Therapeutic Residential Service

**DOI:** 10.1590/0034-7167-2025-0326

**Published:** 2026-07-17

**Authors:** Aldair Weber, Giulia Delfini, Joaquim Manoel de Oliveira Lopes, Vanessa Pellegrino Toledo

**Affiliations:** I Universidade Estadual de Campinas. Campinas, São Paulo, Brazil; II Universidade de Lisboa. Lisboa, Portugal

**Keywords:** Patient Care Team, Aged, Mental Health Services, Deinstitutionalization, Psychiatric Rehabilitation., Grupo de Atención al Paciente, Anciano, Servicios de Salud Mental, Desinstitucionalización, Rehabilitación Psiquiátrica.

## Abstract

**Objectives:**

to understand multidisciplinary team’ experiences in the everyday lifeworld of older residents of Therapeutic Residential Services in a city in the countryside of São Paulo state.

**Methods:**

qualitative research based on Alfred Schutz’s theoretical-methodological framework, conducted through phenomenological interviews with 19 professionals. Analysis revealed three categories that highlight the motivations for care actions.

**Results:**

professionals’ experiences in caring for older adults in a Therapeutic Residential Service are motivated by the bond formed, by biographical aspects, and by existence of challenges faced, such as inherent limitations of care. There is also a search for greater autonomy and for valuing residents’ uniqueness in daily actions.

**Final Considerations:**

observing the experiences that constitute the daily lives of professionals caring for older residents allowed us to recognize their unique and collective needs, and understand the challenges linked to the deinstitutionalization process, in which the Individual Therapeutic Project can guide care actions.

## RESUMEN

**Objetivos:**

comprender las experiencias del equipo multidisciplinario en el entorno cotidiano de ancianos residentes de Servicios Residenciales Terapéuticos en una ciudad del interior del estado de São Paulo.

**Métodos:**

investigación cualitativa basada en el marco teórico-metodológico de Alfred Schutz, realizada mediante entrevistas fenomenológicas a 19 profesionales. El análisis reveló tres categorías que destacan las motivaciones para acciones de cuidado.

**Resultados:**

las experiencias de los profesionales en el cuidado de ancianos en el Servicio Residencial Terapéutico están motivadas por el vínculo afectivo, aspectos biográficos y desafíos experimentados, como las limitaciones propias de la atención. Asimismo, se observa una búsqueda de mayor autonomía y valoración de la singularidad de los residentes en las actividades cotidianas.

**Consideraciones Finales:**

la observación de las experiencias que conforman la vida de los profesionales al cuidar a residentes ancianos nos permitió reconocer necesidades individuales y colectivas, así como comprender los desafíos vinculados al proceso de desinstitucionalización, en el que el Proyecto Terapéutico Individual puede orientar las acciones de cuidado.

## INTRODUCTION

The Brazilian Psychiatric Reform (BPR) broke with the asylum-based model that historically treated people suffering from mental illness through coercive and exclusionary practices^(1)^. Based on the psychosocial paradigm, BPR proposes a care approach centered on individuals and their insertion in the sociocultural context^(1)^. This process culminated in the creation of the Psychosocial Care Network (In Portuguese, *Rede de Atenção Psicossocial* - RAPS), with the objective of structuring and coordinating mental healthcare services, guaranteeing continuity of care within the community and promoting deinstitutionalization^(2)^.

Among the care services provided by the RAPS, Therapeutic Residential Services (TRSs) stand out. These are housing facilities for people experiencing mental distress, discharged from long-term psychiatric hospitalizations, and lacking adequate social or family support^(3)^. These spaces facilitate social reintegration and are organized into two types: I - intended for people in the process of deinstitutionalization; and II - aimed at those with a high level of dependency^(3)^. It is also worth highlighting the recommendation that TRSs be linked to the nearest outpatient mental healthcare service, such as a Psychosocial Care Center (In Portuguese, *Centro de Atenção Psicossocial* - CAPS)^(3)^.

With an interdisciplinary approach, mental healthcare services seek to overcome fragmented and hierarchical models, promoting integrated care flows^(4)^. From this perspective, multidisciplinary teams play a central role in articulating different areas of expertise in the care of people experiencing mental distress^(3,5)^. However, this approach faces challenges, such as work precariousness, the predominance of medical knowledge, and the emphasis on medication logic^(4,5)^, which can compromise individuals’ humanization and autonomy^(4,5)^.

Alongside the challenges faced by teams in RAPS services, within BPR, there is also the need to guarantee mental healthcare for people experiencing psychological distress at any stage of life. Thus, it is important to consider the aging of the Brazilian population^(6)^, in which people aged 65 or over represent 10.9% of the nation^(6)^, presenting new demands for care in the field of mental health, which directly impacts TRSs^(6,7)^. In facilities in Rio de Janeiro, for instance, 41% of residents were over 60 years old, which represents a potential demand for more complex care in these spaces^(7)^. In the deinstitutionalization process, older adults experiencing mental health issues face challenges transitioning to community-based care due to the physical and mental vulnerabilities observed and resulting from institutionalization, with effects such as loss of autonomy and skills for daily living^(7-9)^.

This process still faces challenges globally, such as planning failures, funding shortages, lack of knowledge on how to care for children, absence of adequate community services, and resistance from healthcare professionals, society, and families to adopting a stance of developing autonomy and producing life^(9)^. Although deinstitutionalization policies have transformed mental health support in many countries, such as Brazil^(7)^, their impacts on the aging of people in mental distress are still limited^(8)^. The lack of research focused on this population, coupled with a shortage of specific services and strong social stigma, has led to their invisibility in community-based healthcare strategies^(8)^.

Therefore, research in this area is justified, given that the deinstitutionalization process still faces significant challenges, and that population aging^(6)^ and older adult residents should be considered when planning care actions in TRSs^(7-9)^. As a result, those who benefit from these spaces can develop greater autonomy and communication skills, improved social abilities, and a reduction in psychiatric symptoms^(8)^. In this context, the multidisciplinary team’s work is essential to ensure comprehensive care, promoting psychosocial rehabilitation and quality of life for these individuals^(4,5)^. However, the literature is still limited regarding team daily experiences and the challenges faced in caring for these individuals in TRSs^(8)^.

## OBJECTIVES

To understand multidisciplinary team’s experiences in the everyday lifeworld of older residents of TRSs in a city in the countryside of São Paulo state.

## METHODS

### Ethical aspects

This study was preceded by approval from the *Universidade Estadual de Campinas* Research Ethics Committee. Participants were informed that this was scientific research linked to the principal researcher’s professional practice, aiming to generate reflections to improve care. All received explanations about the research objectives, the procedures adopted, and information regarding voluntary participation, as well as the guarantee of confidentiality of the data obtained. In accordance with Resolution 466/12, interviewees signed the Informed Consent Form and the Authorization for Voice Recording Form before the start of interviews. To preserve anonymity, the statements were identified with the letter “I”, representing “interviewee”, followed by a number corresponding to the sequence in which the interviews were conducted.

### Theoretical-methodological framework

This study is based on the theoretical and methodological approach of Alfred Schutz’s phenomenology, which seeks to understand the phenomenon from the perspective of social action^(10)^. This is conceived as an intentional act, shaped by individual motivations stemming from past (why) and future (for) experiences resulting from subjective interactions established through in-person relationships, which characterizes lifeworld. Thus, action is influenced by biographical trajectory, and these experiences constitute the body of knowledge^(10)^.

### Study design

This is a qualitative study, based on Alfred Schutz’s phenomenology. The research followed COnsolidated criteria for REporting Qualitative research recommendations^(11)^.

### Study setting

The study was developed in a municipality in the countryside of São Paulo state where the RAPS is composed of CAPS III, CAPS Alcohol and Drugs, CAPS Child and Adolescent, psychiatric inpatient beds in a general hospital, TRSs, among others^(12)^. TRSs are linked to CAPS III in the territory where they are located, close to the referral service, as recommended by Ordinance 106 of the Ministry of Health in February 11, 2000^(3)^. Regarding residence structure, type II TRSs have mid-level professionals (health caregiver or monitor) available 24 hours a day and nursing technicians. In type I TRSs, there is a mid-level worker operating only during part of the day^(3)^.

After the study was approved by the institution responsible for TRSs, three CAPS III in the municipality agreed to participate in data collection: two are located in the southwest health district; and one is located in the northwest of the municipality^(12)^. These services are responsible for monitoring different residential units, totaling five type I TRSs, which have a mean of eight residents, and two type II TRSs, which house about ten residents.

It is important to emphasize that the researchers involved in this study work in research, teaching, and mental healthcare in different services within the municipality, ranging from primary care to specialized units. Therefore, both direct and indirect care for older adults is part of their daily practices in these diverse contexts, providing greater familiarity with the topic under investigation.

### Data source

Potential participants in the study included professionals from the multidisciplinary team who work with CAPS III and provide direct care to older adults in TRSs, such as psychologists, nurses, occupational therapists, social workers, physicians, nursing technicians, monitors, and hygiene and housing assistants. Inclusion criteria comprised belonging to the multidisciplinary team that provides care to older adult residents in TRSs, having at least six months of experience in TRS, and not being on vacation or absent from work during the data collection period. Professionals who do not provide direct care to residents, such as pharmacists, pharmacy technicians, hygiene staff, security personnel, and administrative assistants, did not participate in the research.

### Data collection and organization

For this study, snowball sampling was used, a type of non-probabilistic sampling in which one participant indicates another^(13)^, with the first participants from each CAPS being indicated by their respective manager, thus seeking to build a network of trust and credibility throughout the process, in order to avoid possible bias. After authorization for data collection, the institution managing CAPS was notified and instructed the researcher to contact the unit managers, who were referred to as seeds^(13)^, to indicate professionals who they considered to have experiences related to the study’s topic. Subsequently, participants were invited through the researcher’s visits to the service, with the support of managers, who assisted in inviting and scheduling the interviews.

Nineteen people participated in data collection, which took place from January to December 2024, using phenomenological interviews. This method allows individuals to express to the interviewer the meaning they attribute to the experienced phenomenon and the action performed within their context of social interactions^(14)^. Led by the first author, a nurse in the RAPS at the time and experienced in the methodology used, the following trigger questions were employed, “Have you ever cared for an older resident in TRS?”, “Tell me about that care experience”, and “What was your intention in providing that care?”. Based on these questions, further questions were developed to allow the interviewee to delve deeper into the meanings attributed to their experiences.

The interviews took place in various locations, such as CAPS facilities (treatment rooms, studio, among others), in TRSs and in external spaces, according to participants’ preference. They were audio-recorded, with a mean duration of 25 minutes, and there were no repeated interviews. One refusal was recorded for personal reasons, at which time the researcher turned to the seed^(13)^, obtaining a new indication of a participant. Data collection was suspended following a collaborative analysis by the study’s researchers, who realized that the phenomenon had been revealed, the concern had been answered, and no new significant element had emerged in the formation of the categories that represent the typification of the lived experience^(10,15)^.

### Data analysis

Based on the social phenomenology theoretical framework^(10)^, the data were analyzed and organized by the researchers of this study, without the use of software. All interviews conducted were analyzed, following the stages recommended in studies based on Alfred Schutz’s concepts^(10,16)^. Initially, the complete transcription of participants’ statements took place, accompanied by careful reading and rereading of interviews to understand multidisciplinary team professionals’ experiences. This stage was characterized by the suspension of all the researcher’s preconceptions, focusing exclusively on the phenomenon^(10)^.

Subsequently, to understand the social phenomenon, it is necessary to consider the experience’s content, the action subjects, and the historical, social, and situational context, characterizing the subjective experience lived by the people^(10,16)^. The interview captures this experience, and the objective at this stage is to organize the data for logical and systematic analysis. In this way, the passages that express common meanings in the reported experiences are coded into units of subjective meaning, remaining consistent with the meaning that the subjects themselves attribute to their actions^(10)^.

After that, the units of meaning, which highlight the motivations behind interviewees’ actions, were structured into three thematic categories, “Multidisciplinary team’s experiences in the daily care of older residents in the Therapeutic Residential Service”; and “Challenges faced by the multidisciplinary team in caring for older residents in the Therapeutic Residential Service”. Both studies highlighted the “reasons why” behind participants’ lived experiences in the lifeworld, and “Intentionalities in the care of older residents in the Therapeutic Residential Service” addresses the “reasons for” interviewees’ actions. The unveiling of discourses’ subjective meanings was conducted based on social phenomenology and relevant scientific literature^(10)^.

## RESULTS

The study comprised 19 participants: three nurses, two psychologists, two occupational therapists, one social worker, five nursing technicians, five monitors, and one health caregiver/home care assistant. Participants’ ages ranged from 26 to 61 years, all residing in the municipality where the study was conducted. A completed higher education degree is the predominant level of education among them. The mean length of service in TRSs is five years, and their professional trajectories are diverse, including previous experiences as domestic workers, caregivers for older adults, and work in other healthcare services such as psychiatric clinics, inpatient units, and social assistance facilities. The following describes the thematic categories that illustrate participants’ lived experiences in the “us” relationship^(10)^.

### Multidisciplinary team’s experiences in the daily care of older residents in the Therapeutic Residential Service

In their daily work, professionals reflect on old age in the TRS based on their experiences caring for older adults with limitations, such as reduced mobility and communication difficulties.

[...] *we need to think about the therapeutic residency program* [...] *aging, which relates to the older adult population.* (I7)

[...] *she used to walk, then she became very disabled and couldn’t walk anymore. We changed her diapers, provided care, gave her baths, and had to be there to help her bathe* [...]. (I1)

*It involved caring for people with mental health issues who were aging and also had hearing loss.* [...] *the challenge was that communication there was much more intuitive and gestural, and we developed our own way of communicating with them.* (I6)

Another point raised by professionals concerns their first contact with mental health, as they were unfamiliar with TRSs and felt apprehensive about psychiatric patients. In this context, they add that the care to be provided is for older adults experiencing psychological distress, considering them different and requiring support beyond activities of daily living.

*For me, it’s all very new. When I started, it was my first experience with mental health patients. The idea of patients living in residential treatment facilities came about when I joined CAPS.* (I9)

*My fear was of the unknown. The unknown is: psychiatric patients.* (I2)

*I come with experience as a caregiver for older adults.* [...] *it’s different caring for an older adults who has some psychological issue, right? Psychiatric, in this case. It’s not just daily life care. It’s about being able to understand the needs at the end of life, right??* (I17)

In their daily lives, they describe negative aspects of living with an older resident, such as insults, illness, and loss due to death or the need for a transfer of care. Such events cause professionals to feel devastated and worried as well as nostalgic due to the attachment they have developed.

*Every time, I left here feeling devastated, because she has a habit of saying that we steal her private parts* [...] *besides the insults, which are horrible, leaving the house only to be insulted.* (I2)

*When a neighbor gets sick, we get sick too, right? Because we get worried. It’s just that I’m very attached, you know? I’ve become very attached.* (I10)

*Recently, we suffered losses in our homes: one was due to COVID and the other was due to a complication that arose. It was quite a difficult situation.* (I3)

*When you have to transfer a patient, it’s a general struggle for the team. You realize that these are people you’ll miss. The most delicate part is when you become attached to a particular patient* [...]. (I9)

On the other hand, there are positive experiences in daily life with residents, identifying the work as rewarding when providing care in daily practices such as hygiene, medication, feeding, and development of activities, in addition to being able to enter the TRS residents’ world to hear about their experiences.

*It’s a very rewarding job, first of all, right? The care itself is the care with hygiene, right? Care with medication, with food* [...] *and we do some activities with them sometimes.* (I5)

*Because she’s always more in her own world, her speech is disconnected. You can’t really bring it back to reality. And that’s when I step into her conversations. It’s really cool because it’s the moment she returns to reality and can tell me about various experiences she’s had regarding relationships, life experiences, everything, really.* (I19)

From this perspective, professionals’ approach to residents’ daily lives permeates the nuances of living, seeking to mediate relationships, encourage them, understand their needs, and respect individuality and their relationship within the collective of the house.

*You arrive home and see that everyone is in their own room, or one is more depressed, another is more hostile. What’s going on? Why are you feeling this way? You try to get them to interact more, to relax in the house.* (I14)

*We have a very jealous resident who is also older adult. So, you have to be careful even with how you approach her and how you treat her in front of her, because she will almost certainly be attacked afterward* [...]. (I19)

### Challenges faced by the multidisciplinary team in caring for older residents in the Therapeutic Residential Service

The team faces daily challenges when considering networking within an anti-asylum perspective, where the aging of residents is reflected in limitations of care both in CAPS and in the TRS due to difficulties in developing the Singular Therapeutic Project (In Portuguese, *Projeto Terapêutico Singular* - PTS), coupled with a reduced number of staff and professional burnout in the face of care actions that are not implemented.

*There was no way to care for him anymore at CAPS, because people started thinking, “So, are we going to keep the patient in bed? What if he dies at CAPS?”. So, these are issues that the anti-asylum movement hasn’t addressed. We don’t have that discussion.* (I18)

*When we encounter situations that require mobility, for instance, going out for a consultation, or receiving a PTS outside the residence, we already have more difficulties due to transportation and the number of staff members who stay at the house so that another monitor can go out with that person.* (I17)

*While we at CAPS, regarding promotions and staff departures* [...] *we haven’t been able to resolve them. The significant professional burnout has already been considered, including professional retention there, but there’s also this fatigue, you know.* (I8)

Interviewees indicate that financial issues frequently limit the implementation of PTS for older adult residents, in addition to other individual aspects that influence the organization of community space in the TRS. Furthermore, in collective living, the diversity of diagnoses within the same residence makes care more complex, requiring individualized approaches.

*We start thinking about things, but they’re not possible. Not because we don’t want them, but because of financial reasons, sometimes.* [...] *because, at home, they split the expenses, you know. So, these are expenses they have that sometimes make it harder for us to build a more advanced therapeutic plan.* (I17)

*If we could manage to recover the potential of this individual, aspects that were significant throughout their life, I think that, considering the older adult and also the housing situation, it’s not individual housing, it’s collective housing, right? And how often, by being in collective spaces, they need to learn to share and to collectivize things.* (I12)

*Since it’s a residential setting, there’s no patient or resident profile. I think that also makes it difficult. So, we have personality disorders,* [...] *autism spectrum,* [...] *schizophrenia, substance users, all in the same house. All older adults, with physical difficulties, comorbidities. So, we have to individualize the care of each one very much.* (I13)

Professionals reflect on the respectful posture required when entering residents’ homes, as they understand that this is not an extension of CAPS, which highlights the challenge of considering the TRS as both a home and a care space, especially in the face of aging. In this context, professionals question whether practices typical of healthcare services should be incorporated into TRS’ daily routine, as they may mischaracterize it as a residence.

*Whenever we enter the house, we also ask permission, because it’s their home, right? Even though it’s not an extension of CAPS service, you know.* (I3)

*Aging brings us challenges in our thinking. There’s always the division between being a healthcare service and also being a home, right? Including clinical care itself. Is it appropriate for us to take measurements and check vital signs in a home? Is it appropriate to have oxygen available in a home? How do we care for an older adult who sometimes experiences other health problems associated with aging? And how do we manage this within a home?* (I12)

*The home isn’t prepared, it’s not a controlled healthcare facility, because that’s what I think. The older adult is living there, it’s their home. So, we don’t want to turn it into a healthcare facility. And that’s a challenge, understanding that it’s not a healthcare facility, but rather their home. So, how much care can I provide, but also not be constantly checking vital signs?* (I13)

Other challenges are also highlighted, such as allowing residents’ experience to be permeated by the territory dynamics and, furthermore, the articulation of the network beyond mental health needs in order to overcome stigmas.

*Regarding the TRS, I realize it’s a challenge, and we need to coordinate the network, among other things. And to what extent are these people also able to live and participate in the movements within the territory?* (I6)

*A huge challenge is for the care network itself, even in Campinas, to understand that the issue isn’t just psychiatric, because there’s that stigma. That makes our care much more difficult.* (I13)

### Intentionalities in the care of older residents in the Therapeutic Residential Service

Participants desire non-protocol-based care that respects residents’ and TRS community’s uniqueness, and they also intend to promote other activities within the environment with the goal of encouraging residents’ autonomy.

*When I go to the residence* [...] *so that a protocol-driven way of caring is not imposed there, which undermines all those possibilities of construction, which is unique to that group, to that house.* (I6)

*I really miss being able to at least promote other activities within the house* [...] *so, that makes it really difficult, even if you have a body, a number in the house, you can’t do more environmental activities with them, right?* (I8)

*On Thursdays, we have cooking classes here. So, not that you expect something like this, but you see they manage to handle it* [...] *when you go there, and you ask them to peel a potato, put away some green beans. They also put away the groceries* [...] *they manage to pull it off.* (I11)

Finally, professionals demonstrate an intention to improve their actions and are recognized for them, in addition to identifying aspects to be improved in TRS’ daily routine, such as the diet of older adult residents due to clinical comorbidities.

*Did you see what I’m doing out there, the pallets, the flowers? Basically, I’m trying to make a vegetable garden. So, I hope we can keep improving. I always try to improve what I do.* (I15)

*I really enjoy caring for patients. I like to feel that the patient is happy. When giving a bath, especially a mental health patient, they can recognize that care. So, recognition, and it’s very good to feel that the patient is happy and cared for in a way they never had in their life.* (I16)

*I wish things would improve a little more regarding diet, because older adults with diabetes take insulin. I think their diet could be improved. But it’s something that starts, it begins. Then, over time, it gets lost in terms of diet.* (I4)

## DISCUSSION

Understanding multidisciplinary team’s experiences in caring for older adult residents in TRSs is permeated by meanings, actions, attitudes, and relationships established between people and the world in which they live, in accordance with the theoretical assumptions of social phenomenology. The adoption of this research methodology made it possible to access professionals’ experiences and contextualize their intersubjectivities within the lifeworld, a concept that represents the human being in their biographical context constituted by motivations and accumulated knowledge^(10)^.

In their daily care experiences within TRSs, professionals identify the need to consider the aging of residents in these facilities, with their specific care requirements due to reduced mobility, communication difficulties, and other limitations. As a characteristic present in TRSs, human aging at the individual level can be marked by frailty, dependence, and loss of autonomy, which are reflected in care demands^(17)^. Thus, the experience of caregiving is a collective construction based on singularity and interwoven with intersubjective, biographical^(10)^, community, and social aspects^(17)^.

Furthermore, older adults experiencing mental distress present with more complex living and health conditions, requiring adequate recognition of these needs in order to develop specific actions for this population^(18)^. Therefore, professionals in TRSs encounter these older adults and their clinical care demands inherent to old age itself, in addition to many having, in their biographical aspects^(10)^, long periods of institutionalization, as they are discharged from psychiatric hospitals. From a phenomenological perspective, it is perceived that the care provided on a daily basis occurs in the shared encounter of professionals’ and older adult residents’ lifeworld, in which meanings emerge that guide the unique practices in services’ daily routine^(10)^.

Participants also report that, in their experience caring for older adults residing in the TRS, there is initially a fear of psychiatric patients associated with a lack of familiarity with the TRS upon their first contact with this facility, and the need to care for this older adult beyond activities of daily living. The aging Brazilian population, within the context of the country’s deinstitutionalization process, presents persistent challenges, especially regarding older adults’ care units^(6,8)^. Furthermore, TRSs, being recently implemented devices in Brazil^(1)^, still have low social visibility, a perception that is reinforced by participants reporting that they are little known.

In this context, the population’s resistance to the psychiatric deinstitutionalization process not only contributes to the invisibility of existing care strategies^(9)^, but also highlights weaknesses in the training of healthcare professionals, which is often not aligned with BPR principles^(19)^. Thus, in this scenario of older adult residents experiencing psychological distress, it is necessary to consider the need to broaden the approach in training, through the development of studies that can disseminate knowledge about the health scenario and daily life of these residents within the context of the aging process, directly impacting the effectiveness of deinstitutionalization and the strengthening of TRSs^(8,9)^.

The experience of caring for older residents in the TRS is also permeated by negative aspects, such as insults, illness, death, and transfers to other services due to the need for care, leaving behind feelings of longing due to the attachment developed. In the daily routine of mental healthcare services, the creation and establishment of a bond have a great impact on the objectives of care, in which professionals establish an agreement with users on therapeutic goals and tasks to be achieved in the shared world^(10,20)^. Thus, through daily interaction and interaction, professionals develop attachments to residents, which can trigger negative feelings when there is a death or transfer of services. These aspects are anchored in the therapeutic relationship, because when these bonds are broken, professionals are exposed to the experience of emotional distress^(20)^.

At the same time, there are positive experiences lived by participants, expressing that caring for older adult residents is a rewarding job, especially when there is the possibility of learning about their experiences through access to “their world”. In this context, multidisciplinary teamwork in TRSs allows for comprehensive care through the sharing of knowledge and mutual support among professionals^(5)^. This integration strengthens ties with residents, contributing to a more welcoming environment and promoting practices centered on individuals’ uniqueness^(5)^.

In the daily routine of older residents in the TRS, care for the body is also present for professionals, insofar as these aspects may be linked to the historical perspective of fragmented care directed towards physical health^(21)^. However, within the context of deinstitutionalization and the BPR, it is essential to highlight the potential of the multidisciplinary team’s work to change perceptions regarding the professional roles assigned to care^(5,21)^. Joint actions should aim to encourage autonomy and the practice of daily living skills among residents, involving them as protagonists and subjects of choice, capable of leading their own lives within the residential space to the best of their ability^(22)^.

Thus, changes and new perspectives in mental healthcare can be motivated by the multidisciplinary team’s joint actions and related to the rewarding work experience, as expressed by participants in this study. Therefore, professionals’ practice is enhanced when they understand residents’ experiences, recognizing that this is not just a body to be cared for, which makes it possible to build a relationship in the social world based on desires and expectations^(10)^, coinciding with professionals’ statements.

Expanding on the experiences lived in the deinstitutionalization process, among the residents of a TRS, dynamics of coexistence and a way of living are established. The interviewees mention that they mediate this process, valuing individual characteristics and thinking about the construction of a collective space in the house. Reflecting on the living space implies adopting care practices built collectively, sustained in the daily exercise of creating possibilities that promote life, health and well-being, both at the individual and community levels^(23)^, integrating with the recognition of the experiences already lived by residents, which contribute to the finding of meanings in the social world of living^(10)^.

However, when considering the living space in the TRS from a deinstitutionalization perspective, the multidisciplinary team faces challenges, such as limitations of care in both CAPS and TRS due to the aging of residents, highlighting the need for discussions on this issue. This scenario is exacerbated by the RAPS’ structural weaknesses in Brazil, characterized by insufficient service provision and fragmentation, difficulties in communication between points of care, and challenges in implementing intersectoral practices^(2)^. Although the deinstitutionalization model represents a fundamental advance in the field of primary healthcare, there are still significant obstacles, such as stigmatization, underfunding, and, at times, the reproduction of the asylum logic in alternative services^(24)^. This study notes that human aging emerges as a challenge within RAPS services, the effective implementation of the purpose of deinstitutionalization in the BPR, and the current care model. This highlights participants’ concern that this discussion be present in the daily construction of mental healthcare services. In this context, the need to strengthen intersectoral practices, especially those focused on elder care, stands out as a fundamental strategy for the effective implementation of comprehensive care and the continuity of the deinstitutionalization process.

In this context of the RAPS, in alternative services such as TRS, interviewees describe a burnout among professionals due to daily, unsuccessful attempts to build and implement a PTS conceived together with residents. They attribute this to factors such as staff shortages, financial issues, individual needs of each resident, and several psychopathological diagnoses, making care complex. In mental healthcare, the PTS emerges as an important strategy to guarantee aspects such as humanization, comprehensiveness, and equity in the Brazilian context, allowing for the discovery of new approaches to care^(25)^. In the reality of healthcare services, the PTS has been developed in dissonance with what is recommended, highlighting users’ low participation, multidisciplinary knowledge fragmentation, and difficulty in sharing case information for broader discussion^(25)^. The findings of this study support the literature. Therefore, the use of a PTS is recommended as a structuring tool for care, taking into account residents’ individual needs and considering existing difficulties such as staff shortages and financial constraints. Furthermore, developing studies on the effective use of the PTS in TRSs is also an important aspect to be investigated.

With regard to mental healthcare professionals, who share responsibility for the daily process of deinstitutionalization, it is necessary to consider the challenges faced in healthcare services^(26)^. Stress, burnout and difficult interprofessional relationships^(26)^ are shown to be factors that hinder work among mental healthcare professionals, negatively impacting their clinical practices^(27)^, and they are demanding better physical structures, more qualified human resources before entering the service and materials to work with^(28)^. It is reiterated that there are gaps in mental health training, but in order to envision changes in professional practice, innovation and the use of different care tools must be encouraged from within academia^(29)^. Thus, in order to think about a PTS in practice, it is necessary for professionals to find available time for in-person meetings with residents^(10)^, with a view to constituting the care action, in addition to having a structure that makes it possible to achieve the goals proposed in the PTS.

When describing the challenges faced by older residents, participants demonstrate a respectful attitude upon entering the facility, understanding that the TRS is not an extension of CAPS. However, they highlight the challenge of considering the TRS simultaneously as a home for individuals and a space for care, raising questions about the inclusion of practices typical of healthcare services within these facilities. In accordance with legislation regulating TRSs, these facilities must be linked to some healthcare service, emphasizing that, above all, they are living spaces^(3)^. In this environment, due to clinical factors inherent to the aging of residents, the need for other healthcare arises, which seems to challenge the scope of the TRS proposal^(3)^, demanding services with greater support and professional structure. In this context, a lack of reflection in healthcare services on the aging of residents in the TRS is identified, concerning the complexity of the care required and its approach in the context of deinstitutionalization.

From the perspective of caring for residents in their homes, the challenge lies in coordinating care within a network, starting from recognition and experience within the community, as stigma and fragmented care for users experiencing mental distress are still present. The TRS represents the reintegration of institutionalized individuals experiencing mental distress into the community and the prospect of constructing biographical experiences within their lifeworld through newly established relationships^(10)^. However, the fragmentation of mental healthcare remains a daily challenge, where the RAPS structuring and existing intersectoral actions can be ways to sustain, in the long term, the TRS project in the BPR, considering the aging of residents and the success of the deinstitutionalization and psychosocial rehabilitation proposal^(1,2,24)^.

There are also intentions pointed out by interviewees regarding the care they would like to provide, aiming for it to be non-protocol, unique, and to consider other activities within the home environment that encourage autonomy in older adult residents. Considering the TRS, when initiating a process of reclaiming the resident’s citizenship, it is important that healthcare actions reinforce the configuration of a home, not a treatment facility, with a view to producing new biographical experiences and the progressive development of residents towards greater autonomy^(22)^. Furthermore, in the Brazilian context, there are places where a significant portion of residents in TRSs do not regularly attend CAPS, in addition to lacking a PTS and not being included as cases for systematic discussion by the team^(7)^. These observations reinforce the possibility of considering care for older residents through the PTS. However, this requires having diverse resources available to carry out these actions and achieve the intentions expressed by research participants.

Finally, multidisciplinary team professionals seek to improve their actions, appreciate residents’ recognition of the care provided, and look to enhance aspects of daily life at the TRS or even consider proposals for therapeutic activities in the home. One aspect of daily life highlighted by professionals as an expression of the intentionality of their actions is residents’ diet. In line with participants’ expectations, dietary aspects influence older adults’ quality of life, and the social context and difficulties with chewing and swallowing directly interfere with eating behavior^(30)^. Therefore, adopting foods with modified textures is a strategy that can improve intake, and it is recommended to personalize each older adult’s diet according to their independence and level of oral impairment^(30)^. Therefore, it is suggested that this aspect, mentioned by participants as a desire for care, be included in the construction of a PTS with residents, valuing their wishes and seeking longitudinal action from the multidisciplinary team.

There are also other future projects that motivate professionals, such as caring for the exterior of the house by planting flowers or creating a vegetable garden. Such acts of care can contribute to maintaining the TRS as a living space, not characterizing it with the traditional hallmarks of a healthcare service. To strengthen this perspective, it is important that older adult residents actively participate in decision-making about the projects they want to develop, seeking meaning in them, because if these initiatives are solely from the staff, residents may resist being involved as a way of defending their autonomy^(22)^.

### Study limitations

Regarding the study limitations, we highlighted the focus on only a few TRSs in one municipality, not encompassing all existing facilities. There is a need to expand research on this topic, considering aspects of management, work, and living conditions in TRSs in the presence of older adults, as well as the need to reflect on aging within the global movement of psychiatric deinstitutionalization.

### Contributions to the field

This study highlights the actions developed by the multidisciplinary team, which includes nursing professionals, who daily focus on planning the deinstitutionalization process and care for older adult residents in TRSs. Thus, the importance of qualifying these practices as a subsidy for the implementation of the BPR principles and the strengthening of new perspectives in mental health is highlighted. Nursing, due to its close relationship with daily care and as the main workforce in RAPS services, plays a central role in promoting autonomy, monitoring health demands, and improving care practices, which strengthens its role in the multidisciplinary team. As a contribution, this study focuses specifically on older adults in these facilities, given the projected increase in the number of these individuals in care settings in the future. Thus, it reaffirms the importance of teamwork, the expansion of intersectoral actions, and the use of PTS as a fundamental tool to guide comprehensive care centered on residents’ needs.

## FINAL CONSIDERATIONS

In this study, it was possible to construct the lived experience type from thematic categories that highlighted the motivations behind participants’ experiences, as they were permeated by the daily care of TRS older residents in their lifeworld and by the challenges faced, in addition to seeking to maintain the biographical uniqueness of each person in the collective space of the residence.

It was revealed that professionals’ daily lives are permeated by the unknown regarding the TRS in their first contact with mental health, and that, when providing care to older adult residents, negative feelings arise, such as psychiatric patients’ fear. However, through in-person interaction on a daily basis, bonds develop, and the action transforms into positive experiences, making the work rewarding. Furthermore, the biographical aspects and knowledge base of older adult residents should be considered by professionals in their daily work, preserving individual differences within the collective living space.

Between negative and positive experiences, the power of care provided by professionals to older adult residents in TRSs occurs through encounters with their experiences, their subjectivities, and the bonds developed between them, in addition to the importance of the multidisciplinary team’s work in providing comprehensive care. Although fragmented health practices centered on the physical body are still present, the joint action of the team, guided by the principles of deinstitutionalization and BPR, points to a redefinition of professional roles and a paradigm shift in mental healthcare.

The study results also reveal difficulties faced by the multidisciplinary team, where the aging of residents in the TRS is seen as a challenge given the limitations of care within RAPS facilities, leading to burnout and professional demotivation among the teams. In this context, the PTS emerges as a structuring tool for considering older adults residents’ challenges and individual needs, as well as the residence dynamics. Another finding is the concern about not converting the living space in the TRS into a healthcare service, maintaining the purpose of a facility created from a deinstitutionalization perspective, but which requires further reflection on the care of older residents in this space.

Promoting autonomy and valuing older residents’ uniqueness in the TRS are presented as perspectives that guide the care provided by professionals, whose actions seek to improve every day, in a continuous movement of constructing the living space, the TRS as a device for deinstitutionalization and the implementation of BPR principles.

## Data Availability

The research data are available only upon request.
