# Supplementary material for: Multidisciplinary team’s experiences caring for older adults in the Therapeutic Residential Service
Source: Rev Bras Enferm. 2026 Jul 17;79:e20250326. doi: 10.1590/0034-7167-2025-0326 (PMC13379238; doi:10.1590/0034-7167-2025-0326)
Supplement: Translation

# Translation: Vivências da equipe multiprofissional no cuidado ao idoso no Serviço Residencial Terapêutico

**DOI:** 10.1590/0034-7167-2025-0326pt

**Authors:** Aldair Weber, Giulia Delfini, Joaquim Manoel de Oliveira Lopes, Vanessa Pellegrino Toledo

**Affiliations:** I Universidade Estadual de Campinas. Campinas, São Paulo, Brasil; II Universidade de Lisboa. Lisboa, Portugal

**Keywords:** Equipe Multiprofissional, Idoso, Serviço Residencial Terapêutico em Saúde Mental, Desinstitucionalização, Reabilitação Psiquiátrica.

## Abstract

**Objetivos:**

compreender as vivências da equipe multiprofissional no mundo-vida cotidiano dos moradores idosos de Serviços Residenciais Terapêuticos em uma cidade do interior paulista.

**Métodos:**

pesquisa qualitativa baseada no referencial teórico-metodológico de Alfred Schutz, realizada por meio de entrevistas fenomenológicas com 19 profissionais. A análise desvelou três categorias que evidenciam motivações da ação do cuidado.

**Resultados:**

as vivências dos profissionais no cuidado ao idoso no Serviço Residencial Terapêutico são motivadas pelo vínculo, por aspectos biográficos e pela existência de desafios vividos, como os próprios limites do cuidado. Há também a busca por maior autonomia e pela valorização da singularidade do morador nas ações diárias.

**Considerações Finais:**

a observação das experiências que constituem as vivências dos profissionais ao cuidarem do morador idoso permitiu reconhecer as necessidades singulares e coletivas, bem como compreender os desafios vinculados ao processo de desinstitucionalização, no qual o Projeto Terapêutico Singular pode nortear as ações do cuidado.

## INTRODUÇÃO

A Reforma Psiquiátrica Brasileira (RPB) rompeu com o modelo manicomial-asilar que historicamente tratava pessoas em sofrimento psíquico por meio de práticas coercitivas e excludentes^(1)^. Fundamentada no paradigma psicossocial, a RPB propõe uma abordagem de cuidado centrada no sujeito e em sua inserção no contexto sociocultural^(1)^. Tal processo culminou na criação da Rede de Atenção Psicossocial (RAPS), com o objetivo de estruturar e articular os serviços de saúde mental, garantindo a continuidade do cuidado no território e promovendo a desinstitucionalização^(2)^.

Entre os dispositivos de cuidado da RAPS, destacam-se os Serviços Residenciais Terapêuticos (SRTs), moradias de pessoas em sofrimento psíquico, egressas de longas internações psiquiátricas e que não possuem suporte social nem familiar adequado^(3)^. Esses espaços viabilizam a reinserção social, e são organizados em duas tipologias: I - destinada a pessoas em processo de desinstitucionalização; e II - voltada àquelas com elevado nível de dependência^(3)^. Salienta-se, ainda, a recomendação de que os SRTs estejam vinculados ao serviço ambulatorial de saúde mental mais próximo, como um Centro de Atenção Psicossocial (CAPS)^(3)^.

Com uma abordagem interdisciplinar, os serviços de saúde mental buscam superar modelos fragmentados e hierarquizados, promovendo fluxos assistenciais integrados^(4)^. Nessa perspectiva, as equipes multiprofissionais desempenham um papel central ao articular diferentes saberes no cuidado às pessoas em sofrimento psíquico^(3,5)^. No entanto, tal abordagem enfrenta desafios, como a precarização do trabalho, o predomínio do saber médico e a ênfase na lógica medicamentosa^(4,5)^, que podem comprometer a humanização e autonomia dos indivíduos^(4,5)^.

Em conjunto aos desafios vividos pelas equipes nos serviços da RAPS, no âmbito da RPB, há também a necessidade de se garantir o cuidado em saúde mental pessoa em sofrimento psíquico em qualquer ciclo da vida. Assim, é importante considerar o envelhecimento da população brasileira^(6)^, em que as pessoas com 65 anos ou mais correspondem a 10,9% da nação^(6)^, apresentando novas demandas de cuidados ao campo da saúde mental, o que impacta diretamente os SRTs^(6,7)^. Em dispositivos do Rio de Janeiro, por exemplo, 41% dos moradores possuíam mais de 60 anos, o que representa uma possível demanda de cuidados mais complexos nesses espaços^(7)^. No processo de desinstitucionalização, os indivíduos idosos em sofrimento psíquico vivenciam desafios na transição para dispositivos na comunidade devido à vulnerabilidade física e mental observadas e resultantes da institucionalização, com efeitos como perda de autonomia e de habilidades para a vida diária^(7-9)^.

Esse processo ainda enfrenta desafios em nível global, como falhas no planejamento, escassez de financiamento, falta de conhecimento em como cuidar, ausência de serviços comunitários adequados, e resistência de profissionais de saúde, da sociedade e de familiares em adotar uma postura de desenvolvimento de autonomia e produção de vida^(9)^. Embora as políticas de desinstitucionalização tenham transformado o suporte à saúde mental em muitos países, tal como no Brasil^(7)^, seus impactos sobre o envelhecimento de pessoas em sofrimento psíquico ainda são limitados^(8)^. A falta de pesquisas voltadas para essa população, somada à carência de serviços específicos e ao forte estigma social, tem levado à sua invisibilidade nas estratégias de atenção comunitária à saúde^(8)^.

Portanto, justifica-se o desenvolvimento de pesquisas neste âmbito, tendo em vista que o processo de desinstitucionalização ainda enfrenta desafios significativos, sendo que o envelhecimento populacional^(6)^ e os moradores idosos devem ser fatores considerados para planejar as ações de cuidados nos SRTs^(7-9)^. Como resultado, aqueles que se beneficiam desses espaços podem desenvolver maior autonomia e capacidade de comunicação, melhora nas habilidades no mundo social, e redução dos sintomas psiquiátricos^(8)^. Nesse contexto, a atuação da equipe multiprofissional é essencial para garantir um cuidado integral, promovendo a reabilitação psicossocial e qualidade de vida desses indivíduos^(4,5)^. No entanto, a literatura ainda é limitada quanto às vivências das equipes no cotidiano e aos desafios enfrentados no cuidado a essas pessoas nos SRTs^(8)^.

## OBJETIVOS

Compreender as vivências da equipe multiprofissional no mundo-vida cotidiano dos moradores idosos nos SRTs em uma cidade do interior paulista.

## MÉTODOS

### Aspectos éticos

Este estudo foi precedido da aprovação do Comitê de Ética em Pesquisa da Universidade Estadual de Campinas. Os participantes foram informados de que se tratava de pesquisa científica vinculada à prática profissional do pesquisador principal, visando gerar reflexões para qualificar o cuidado. Todos receberam esclarecimentos sobre os objetivos da pesquisa, os procedimentos adotados e as informações referentes à participação voluntária, bem como à garantia de sigilo dos dados obtidos. Conforme a Resolução nº 466/12, os entrevistados assinaram o Termo de Consentimento Livre e Esclarecido e o Termo de Autorização para Gravação da Voz antes do início das entrevistas. Para preservar o anonimato, os relatos foram identificados com a letra “E”, representando “entrevistado”, seguida de um número correspondente à sequência em que as entrevistas foram realizadas.

### Referencial teórico-metodológico

Estudo fundamentado na abordagem teórico-metodológica, da fenomenologia de Alfred Schutz, que busca compreender o fenômeno a partir da ação social^(10)^. Essa é concebida como um ato intencional, moldado pelas motivações individuais de vivências passadas (motivos por que) e futuras (motivos para) resultantes das interações subjetivas estabelecidas, por meio da relação face a face, o que caracteriza o mundo-vida. Assim, a ação é influenciada pela trajetória biográfica, e essas vivências constituem o acervo de conhecimento^(10)^.

### Tipo de estudo

Trata-se de estudo qualitativo, amparado na fenomenologia de Alfred Schutz. Para o desenvolvimento da pesquisa, foram seguidas as recomendações do *COnsolidated criteria for REporting Qualitative research* ^(11)^.

### Cenário do estudo

O estudo foi desenvolvido em um município do interior paulista onde a RAPS é composta por CAPS III, CAPS Álcool e Drogas, CAPS Infantojuvenil, leitos de internação psiquiátrica em hospital geral, SRTs, entre outros^(12)^. Os SRTs são vinculados aos CAPS III do território onde estão inseridos, próximos ao serviço de referência, conforme a recomendação da Portaria nº 106 do Ministério da Saúde, de 11 de fevereiro de 2000^(3)^. Quanto à estrutura das residências, os SRTs tipo II contam com profissionais de nível médio (cuidador em saúde ou monitor) disponíveis 24 horas por dia e técnicos de enfermagem. Já nos SRTs tipo I, há um trabalhador de nível médio atuando apenas em um período do dia^(3)^.

Após a aprovação do estudo pela instituição responsável pelos SRTs, três CAPS III do município aceitaram participar da coleta de dados: dois estão localizados no distrito de saúde sudoeste; e um está localizado no noroeste do município^(12)^. Esses serviços são responsáveis pelo acompanhamento de diferentes unidades residenciais, totalizando cinco SRTs tipo I, que possuem, em média, oito residentes, e dois da tipologia II, que abrigam cerca de dez moradores.

É importante ressaltar que os pesquisadores envolvidos neste estudo atuam em pesquisa, ensino e assistência em saúde mental em diferentes serviços do município, desde a atenção primária até unidades especializadas. Assim, tanto o cuidado direto quanto o indireto com as pessoas idosas fazem parte de suas práticas cotidianas nesses diversos contextos, proporcionando uma maior proximidade com a temática investigada.

### Fonte de dados

Como possíveis participantes do estudo, tem-se os profissionais da equipe multiprofissional que atuam junto aos CAPS III e desenvolvem cuidados diretos aos idosos dos SRTs, tais como psicólogos, enfermeiros, terapeutas ocupacionais, assistentes sociais, médicos, técnicos de enfermagem, monitores, e auxiliares de higiene e de moradia. Os critérios de inclusão foram pertencer à equipe multiprofissional que presta cuidados a moradores idosos dos SRTs, ter pelo menos seis meses de experiência no SRT e no período da coleta de dados não estar de férias ou afastado do trabalho. Não participaram da pesquisa os profissionais que não prestam cuidados diretos aos moradores, tais como farmacêuticos, técnicos de farmácia, equipe de higiene, serviço de vigilância e auxiliares administrativos.

### Coleta e organização de dados

Para este estudo, foi utilizada a amostragem em bola de neve, tipo de amostragem não probabilística, em que um participante indica o outro^(13)^, sendo que os primeiros participantes de cada CAPS foram indicados pelo respectivo gestor, buscando, assim, construir uma rede de confiança e credibilidade ao longo do processo, a fim de evitar um possível viés. Após a liberação para a coleta de dados, a instituição gerenciadora dos CAPS foi notificada e orientou o pesquisador a se direcionar aos gestores das unidades, sendo estes referenciados como sementes^(13)^, para indicar profissionais que consideravam ter vivências relacionadas à temática do estudo. Em seguida, os participantes foram convidados por meio das visitas do pesquisador ao serviço, com o apoio dos gestores, que auxiliaram no convite e agendamento das entrevistas.

Participaram 19 pessoas da coleta de dados, realizada de janeiro a dezembro de 2024, por meio da entrevista fenomenológica, que permite que o indivíduo expresse ao entrevistador o significado atribuído ao fenômeno vivenciado e à ação realizada em seu contexto de interações sociais^(14)^. Conduzidas pelo primeiro autor, enfermeiro da RAPS na época e com experiência na metodologia utilizada, foram utilizadas as seguintes questões disparadoras: “Você já cuidou de algum morador idoso no SRT?”, “Conte-me como foi esse cuidado” e “Qual sua intenção ao realizar esse cuidado?”. A partir dessas perguntas, prosseguiu-se à elaboração de outras questões que permitiram ao entrevistado aprofundar os sentidos atribuídos às suas vivências.

As entrevistas ocorreram em locais diversos, como as dependências do CAPS (salas de atendimento, ateliê, entre outros), nos SRTs e em espaços externos, conforme preferência do participante. Foram gravadas em áudio, com duração média de 25 minutos, e não houve entrevistas repetidas. Registrou-se uma recusa por motivos pessoais, ocasião na qual o pesquisador se voltou à semente^(13)^, obtendo nova indicação de participante. A coleta de dados foi suspensa a partir da análise colaborativa dos investigadores do estudo ao perceberem que o fenômeno havia sido desvelado, a inquietação havia sido respondida, e nenhum novo elemento significativo havia surgido na formação das categorias que representam a tipificação da experiência vivida^(10,15)^.

### Análise de dados

Amparado no referencial teórico da fenomenologia social^(10)^, os dados foram analisados e organizados pelos pesquisadores deste estudo, sem uso de *software*. Todas as entrevistas realizadas foram analisadas, observando as etapas recomendadas nos estudos fundamentados nos conceitos de Alfred Schutz^(10,16)^. Inicialmente, ocorreu a transcrição completa dos depoimentos dos participantes, acompanhada da leitura e releitura atenta das entrevistas para apreender o significado das vivências dos profissionais da equipe multiprofissional, sendo essa etapa caracterizada pela suspensão de todos os pré-julgamentos do pesquisador, voltando-se exclusivamente ao fenômeno^(10)^.

Em seguida, para compreender o fenômeno social, é preciso considerar o conteúdo da experiência, os sujeitos da ação, e o contexto histórico, social e situacional, caracterizando a experiência subjetiva vivida pelas pessoas^(10,16)^. A entrevista capta essa vivência, e o objetivo nesta etapa é organizar os dados para serem analisados de forma lógica e sistemática. Dessa forma, os trechos que expressam sentidos comuns nas vivências relatadas são codificados em unidades de sentidos subjetivos, mantendo-se coerente com o sentido que os próprios sujeitos atribuem às suas ações^(10)^.

Após isso, as unidades de sentido, que evidenciam as motivações das ações dos entrevistados, foram estruturadas em três categorias temáticas: “Vivências da equipe multiprofissional no cuidado diário ao morador idoso no Serviço Residencial Terapêutico”; e “Desafios vividos pela equipe multiprofissional no cuidado ao morador idoso do Serviço Residencial Terapêutico”. Ambas destacaram os “motivos porque” da experiência vivida no mundo-vida dos participantes, e “Intencionalidades no cuidado ao morador idoso no Serviço Residencial Terapêutico” versa sobre os “motivos para” da ação dos entrevistados. O desvelamento dos significados subjetivos dos discursos foi conduzido com base na fenomenologia social e na literatura científica pertinente^(10)^.

## RESULTADOS

O estudo foi composto por 19 participantes, sendo três enfermeiros, dois psicólogos, dois terapeutas ocupacionais, um assistente social, cinco técnicos de enfermagem, cinco monitores e um cuidador em saúde/auxiliar de moradia. A faixa etária dos participantes varia entre 26 e 61 anos, todos residentes no município onde o estudo foi realizado. O ensino superior completo constitui o grau de escolaridade predominante entre eles. O tempo médio de atuação nos SRTs é de cinco anos, e suas trajetórias profissionais são diversas, incluindo experiências anteriores, como empregada doméstica, cuidador de idosos e, ainda, atuação em outros serviços de saúde, como clínicas psiquiátricas, unidades de internação e equipamentos da assistência social. A seguir, encontram-se descritas as categorias temáticas que ilustram o tipo vivido das ações dos participantes na relação “nós”^(10)^.

### Vivências da equipe multiprofissional no cuidado diário ao morador idoso no Serviço Residencial Terapêutico

No cotidiano, os profissionais refletem sobre a velhice no SRT a partir das vivências caracterizadas por cuidados a idosos com limitações, como mobilidade reduzida e dificuldade de comunicação.

[...] *a gente pensar no programa da residência terapêutica* [...] *o envelhecimento, que tem a ver com o público idoso.* (E7)

[...] *ela andava, depois ficou bem incapacitada, não conseguia andar. A gente trocava fralda, dava os cuidados, dava banho, tinha que acompanhar no banho* [...]. (E1)

*Era cuidar de pessoas com questões de saúde mental num processo de envelhecimento e ainda com um déficit auditivo.* [...] *com o desafio de que a comunicação ali era muito mais intuitiva, gestual e que a gente foi criando ali um jeito de se comunicar com elas.* (E6)

Outro apontamento dos profissionais versa sobre o seu primeiro contato com a saúde mental, pois desconheciam sobre os SRTs e sentiram medo em relação aos pacientes psiquiátricos. Nesse contexto, acrescentam que o cuidado a ser realizado é com idosos em sofrimento psíquico, considerando-os diferentes, necessitando ir para além do suporte às atividades de vida diária.

*Para mim, é tudo muito novo. Quando entrei, foi minha primeira experiência com paciente da saúde mental. A ideia dos pacientes moradores de SRT veio quando entrei no CAPS.* (E9)

*O meu medo era do desconhecido. O desconhecido é: pacientes psiquiátricos.* (E2)

*Eu venho com experiência de cuidadora de idosos.* [...] *é diferente um cuidado com idoso que tem alguma questão psicológica, né? Psiquiátrica, no caso. Não é só o cuidado de vida diária. É um cuidado de você conseguir compreender as necessidades do final da vida, né?* (E17)

No dia a dia, descrevem aspectos negativos da vivência com o morador idoso, como xingamentos, adoecimento e perda por falecimento ou pela necessidade de transferência de cuidados. Tais acontecimentos fazem com que os profissionais se sintam arrasados e preocupados, além de sentirem saudades devido ao apego desenvolvido.

*Toda vez, eu saía daqui muito arrasada, porque ela tem hábito de falar que a gente rouba partes íntimas dela* [...] *fora os xingamentos, que é horrível, a gente sair de casa para ser xingada.* (E2)

*Quando morador fica doente, a gente fica doente também, né? Porque fica preocupado. É que eu sou muito apegada, né? Eu me apeguei muito.* (E10)

*Recentemente, a gente teve perdas nas nossas moradias: uma foi de COVID e a outra foi uma complicação que surgiu. Foi algo bem complicado.* (E3)

*Você tem que transferir um paciente, é um sofrimento geral da equipe. Você vê que são pessoas que deixam saudades. A parte mais delicada é quando você se apega a determinado paciente* [...]. (E9)

Por outro lado, há experiências positivas no cotidiano com os moradores, identificando o trabalho como gratificante ao prestar o cuidado em práticas diárias como higiene, medicação, alimentação e desenvolvimento de atividades, além de conseguirem entrar no mundo do morador do SRT para ouvir sobre suas vivências.

*É um trabalho muito gratificante, em primeiro lugar, né? O cuidado em si é o cuidado com a higiene, né? Cuidado com a medicação, com a alimentação* [...] *e a gente faz algumas atividades, às vezes, com eles.* (E5)

*Por ela estar sempre mais no mundo dela, são falas desconexas. Você não consegue trazer muito pra realidade. E aí eu entro dentro dessas falas. É muito legal porque é o momento que ela volta pra realidade e consegue me contar várias experiências que teve sobre relacionamentos, sobre vivências, enfim, de tudo.* (E19)

Nessa perspectiva, o manejo dos profissionais no cotidiano dos moradores permeia as nuances do morar, ao buscar mediar as relações de convivência, estimulá-los e entender suas demandas, assim como respeitar a individualidade e sua relação no coletivo da casa.

*Você chega na casa, vê que está cada um no quarto, ou um está mais deprimido, outro está mais hostil. O que está acontecendo? Por que você está assim? Você tenta puxar eles para interagir mais, para descontrair na casa.* (E14)

*A gente tem uma moradora que é muito ciumenta, que também é idosa. Então, você tem que tomar cuidado até com a forma que aborda e o tratamento que faz na frente da moradora, porque, com certeza, ela vai ser agredida depois* [...]. (E19)

### Desafios vividos pela equipe multiprofissional no cuidado ao morador idoso do Serviço Residencial Terapêutico

A equipe se depara com desafios no cotidiano quando considerado o trabalho em rede na perspectiva antimanicomial, no qual o envelhecimento dos moradores reflete em limites de cuidado tanto no CAPS quanto no SRT devido às dificuldades no desenvolvimento do Projeto Terapêutico Singular (PTS), somadas ao reduzido número de funcionários e ao esgotamento profissional diante de ações de cuidado que não são implementadas.

*Não tinha mais como cuidar dele lá no CAPS, porque as pessoas começaram a pensar: “E aí, a gente vai ficar com o morador no leito? E se ele morrer no CAPS?”. Então, são questões que a luta antimanicomial não chegou. A gente não tem essa discussão.* (E18)

*Quando esbarra em coisas que necessitam de mobilidade, por exemplo, saída para consulta, PTS fora da moradia, a gente já tem mais dificuldade por conta de transporte, da quantidade de funcionários que ficam na casa para que outro monitor consiga sair com essa pessoa.* (E17)

*Enquanto CAPS, essa questão da promoção, de saídas* [...] *a gente não vai conseguindo. O desgaste profissional importante já foi pensado, inclusive a fixação de profissional lá, mas também tem esse cansaço, né.* (E8)

Os entrevistados apontam que questões financeiras frequentemente limitam a implementação do PTS dos moradores idosos, além de outros aspectos individuais que influenciam a organização do espaço comunitário no SRT. Além disso, na convivência coletiva, a diversidade de diagnósticos na mesma residência torna o cuidado mais complexo, exigindo abordagens individualizadas.

*A gente vai pensando em coisas, mas que não são possíveis. Não porque a gente não quer, mas por conta das coisas financeiras mesmo, às vezes.* [...] *porque, na casa, eles dividem os gastos, né. Então, são gastos que eles têm e que, às vezes, dificulta mais a gente conseguir construir um projeto terapêutico mais avançado.* (E17)

*Se a gente conseguisse resgatar as potencialidades desse sujeito, questões que foram significativas ao longo da sua vida, acho que pensando no idoso e também numa moradia, não é uma moradia individual, é uma moradia coletiva, né? E o quanto, muitas vezes, por estar em espaços coletivos, precisa aprender a dividir, a coletivizar questões.* (E12)

*Como é moradia, não tem perfil de paciente, de morador. Eu acho que isso dificulta também. Então, nós temos transtornos de personalidade,* [...] *espectro autista,* [...] *esquizofrenia, usuários de substâncias, tudo na mesma casa. Todos idosos, com dificuldades físicas, comorbidades. Então, a gente tem que individualizar muito o cuidado de cada um.* (E13)

Os profissionais refletem sobre a postura respeitosa ao adentrar a casa dos moradores, pois compreendem que essa não é uma extensão do CAPS, o que caracteriza um desafio de pensar o SRT como lar e espaço de cuidados ao mesmo tempo, especialmente diante do envelhecimento. Nesse contexto, os profissionais questionam se práticas típicas de serviços de saúde devem ser incorporadas no cotidiano do SRT, pois podem descaracterizá-lo como residência.

*Sempre que a gente vai entrar na casa, também, a gente pede licença, porque ali é a casa deles, né? Apesar de não ser uma extensão do serviço, né, do CAPS.* (E3)

*Envelhecimento, isso vai nos trazendo desafios de pensar. Sempre a divisão em ser um serviço de saúde, mas também ser uma casa, né? Inclusive de cuidados clínicos mesmo. Cabe a gente fazer aferições e sinais dentro de uma casa? Cabe ter o oxigênio dentro da casa? Como cuidar de um senhor onde, às vezes, vai tendo outros processos de adoecimento associado ao seu envelhecimento? E como a gente cuida isso dentro de uma casa?* (E12)

*A casa não é preparada, não é um serviço de saúde controlado, porque eu acho que é isso. O idoso, ele está morando, é a casa dele. Então, a gente não quer transformar isso em um serviço de saúde. E isso é uma dificuldade, a gente entender que não é um serviço de saúde, e sim a casa dele. Então, o quanto eu consigo prestar esse cuidado, mas também não ficar aferindo sinais vitais o tempo todo.* (E13)

Outros desafios também são apontados, como permitir que a vivência do morador esteja permeada pelas dinâmicas do território e, ainda, a articulação da rede para além das necessidades de saúde mental com vistas a superar estigmas.

*Sobre a SRT, percebo que é um desafio, e a gente precisa articular a rede, inclusive. E o quanto que essas pessoas também conseguem viver e fazer parte dos movimentos do território.* (E6)

*Um grandíssimo desafio é a própria rede de cuidado, de Campinas mesmo, entender que não é só psiquiátrica a questão, porque tem aquele estigma. Isso dificulta muito nosso cuidado.* (E13)

### Intencionalidades no cuidado ao morador idoso no Serviço Residencial Terapêutico

Os participantes anseiam um cuidado não protocolar e que respeite a singularidade do morador e do coletivo do SRT, assim como intencionam promover outras atividades na ambiência com o objetivo de estimular a autonomia dos moradores.

*Quando vou para a moradia* [...] *para que não seja impresso ali um modo de cuidar protocolar que mine todas essas possibilidades de construção, que é singular, daquele grupo, daquela casa.* (E6)

*Eu sinto muita falta de conseguir pelo menos promover outras atividades dentro da casa* [...] *então, isso vai dificultando de fato, mesmo que você tenha um corpo, um número na casa, você não consegue fazer uma atividade mais de ambiência com eles, né.* (E8)

*De quinta-feira, a gente tem culinária aqui. Então, não que você espera algo assim, mas você vê conseguindo dar conta ali* [...] *quando você vai lá, e você pede pra eles descascar uma batata, guardar um varejão. Eles também que guardam a compra* [...] *conseguem desenrolar.* (E11)

Por fim, os profissionais demonstram a intencionalidade de melhorar suas ações e são reconhecidos por elas, além de identificarem aspectos a serem incrementados no cotidiano do SRT, como a alimentação dos moradores idosos devido às comorbidades clínicas.

*Você viu o que estou fazendo aí fora, os paletes, as flores. No fundo, estou tentando fazer uma horta. Então, espero que a gente continue melhorando. Procuro sempre melhorar no que faço.* (E15)

*Eu gosto muito de cuidar. Eu gosto de sentir que o paciente está feliz. Quando faz um banho, ainda mais o paciente de saúde mental, ele consegue reconhecer esse cuidado. Então, reconhecimento, e é muito bom sentir que o paciente está feliz e cuidado do que ele nunca teve na vida.* (E16)

*Gostaria que melhorasse um pouquinho mais com relação à alimentação, porque os idosos com diabetes tomam insulina. Eu acho que a alimentação poderia ser melhorada. Mas é uma coisa que se inicia, começa. Depois, ao longo do tempo, ela vai se perdendo com relação à alimentação.* (E4)

## DISCUSSÃO

A compreensão das vivências da equipe multiprofissional no cuidado aos moradores idosos nos SRTs está permeada por significados, ações, atitudes e relações estabelecidas entre as pessoas e o mundo em que vivem, consoante aos pressupostos teóricos da fenomenologia social. A adoção dessa metodologia de pesquisa possibilitou acessar a experiência dos profissionais e contextualizar suas intersubjetividades no mundo-vida, conceito que representa o ser humano em seu cenário biográfico constituído pelas motivações e acervo de conhecimento^(10)^.

Em suas vivências de cuidado no cotidiano dos SRTs, os profissionais identificam a necessidade de olhar para o envelhecimento dos moradores nesses dispositivos, com a demanda de cuidados específicos devido à mobilidade reduzida, à dificuldade de comunicação e a outras limitações. Como característica presente nos SRTs, o envelhecimento humano no âmbito individual pode ser marcado por relações de fragilização, dependência e perda de autonomia, com reflexo nas demandas de cuidado^(17)^. Assim, a experiência de cuidar é uma construção coletiva baseada na singularidade e entrelaçada por aspectos intersubjetivos, biográficos^(10)^, comunitários e sociais^(17)^.

Ademais, os idosos em sofrimento psíquico apresentam maior complexidade das condições de vida e de saúde, requerendo um adequado reconhecimento dessas necessidades para pensar ações específicas a esse público^(18)^. Portanto, os profissionais nos SRTs se deparam com esses idosos e suas demandas de cuidado clínico inerentes à própria velhice, além de muitos terem, em seus aspectos biográficos^(10)^, os longos períodos de institucionalização, por serem egressos de hospitais psiquiátricos. Sob a perspectiva fenomenológica, percebe-se que o cuidado realizado no dia a dia ocorre no encontro compartilhado do mundo-vida dos profissionais e dos moradores idosos, em que emergem sentidos que orientam as práticas singulares no cotidiano dos serviços^(10)^.

Os participantes também relatam que, na experiência de cuidado com idosos que residem nos SRTs, inicialmente, há o medo do paciente psiquiátrico associado ao desconhecimento do SRT, na ocasião de seu primeiro contato com esse dispositivo, e a necessidade de cuidar desse idoso para além das atividades de vida diária. O envelhecimento populacional brasileiro, no contexto do processo de desinstitucionalização no país, impõe desafios que persistem, especialmente no que se refere às demandas de cuidado a pessoas idosas^(6,8)^. Além disso, os SRTs, por serem dispositivos de implementação recente no Brasil^(1)^, ainda apresentam baixa visibilidade social, percepção que é reforçada pelos participantes ao relatarem que são pouco conhecidos.

Nesse contexto, a resistência da população ao processo de desinstitucionalização psiquiátrica não apenas contribui para invisibilidade das estratégias de cuidado existentes^(9)^, mas também evidencia as fragilidades na formação dos profissionais de saúde que, muitas vezes, não está alinhada aos princípios da RPB^(19)^. Assim, neste cenário de residentes idosos em sofrimento psíquico, é preciso considerar a necessidade de ampliar a abordagem na formação, através do desenvolvimento de estudos que possam divulgar o conhecimento sobre o cenário de saúde e o cotidiano de vida desses moradores no âmbito do processo de envelhecimento, repercutindo diretamente na efetivação da desinstitucionalização e no fortalecimento dos SRTs^(8,9)^.

A vivência de cuidado ao morador idoso no SRT também é permeada por aspectos negativos, como xingamentos, adoecimento, falecimento e transferências para outros serviços devido à necessidade de cuidados, deixando saudades em virtude do apego desenvolvido. No cotidiano dos serviços de saúde mental, a criação e o estabelecimento do vínculo têm grande impacto nos objetivos do cuidado, em que o profissional estabelece com o usuário um acordo sobre metas e tarefas terapêuticas a serem alcançadas no mundo compartilhado^(10,20)^. Assim, na vinculação e convivência cotidiana, os profissionais desenvolvem apego aos moradores, o que desperta sentimentos negativos quando há falecimento ou transferência de serviços, sendo esses aspectos ancorados na relação terapêutica, pois, quando esses laços são rompidos, os profissionais se expõem à experiência de sofrimento emocional^(20)^.

Ao mesmo tempo, há experiências positivas vividas pelos participantes, expressando que o cuidado aos moradores idosos é um trabalho gratificante, principalmente quando há possibilidade de conhecer suas vivências por meio do acesso ao “mundo deles”. Nesse contexto, o trabalho em equipe multiprofissional nos SRTs permite o cuidado integral por meio do compartilhamento de saberes e do apoio mútuo entre os profissionais^(5)^. Essa integração fortalece vínculos com os moradores, contribuindo para um ambiente mais acolhedor e promovendo práticas centradas na singularidade da pessoa^(5)^.

No cotidiano do morador idoso no SRT, para os profissionais, os cuidados com o corpo também se fazem presentes na medida em que esses aspectos podem estar vinculados à perspectiva histórica do cuidado fragmentado e direcionado à saúde física^(21)^. Porém, no âmbito da desinstitucionalização e da RPB, é essencial apontar a potencialidade do trabalho da equipe multiprofissional para as mudanças de percepções em relação aos papéis profissionais designados ao cuidado^(5,21)^. As ações em conjunto devem buscar o estímulo à autonomia e o exercício das habilidades de vida diária dos moradores, envolvendo-os como protagonistas e sujeitos de escolhas, capazes de conduzir, na medida de suas possibilidades, suas próprias vidas no espaço residencial^(22)^.

Assim, as mudanças e novas perspectivas no cuidado em saúde mental podem ser motivadas pelas ações em conjunto da equipe multiprofissional e relacionadas à experiência gratificante de trabalho, como expresso pelos participantes deste estudo. Diante disso, a prática dos profissionais é potencializada ao conhecerem as vivências do morador, compreendendo que este não é somente um corpo a ser cuidado, o que possibilita construir uma relação no mundo social a partir dos desejos e expectativas^(10)^, coadunando com os relatos dos profissionais.

Ampliando as experiências vividas no processo de desinstitucionalização, entre os moradores de um SRT, estabelecem-se dinâmicas de convivência e um modo de morar. Os entrevistados mencionam que realizam a mediação desse processo, valorizando as características individuais e pensando na construção do espaço coletivo da casa. Refletir sobre o espaço do morar implica adotar práticas de cuidado construídas de forma coletiva, sustentadas no exercício cotidiano de criar possibilidades que promovam vida, saúde e bem-estar, tanto em nível individual quanto em nível comunitário^(23)^, integrando-se ao reconhecimento das experiências já vividas pelos moradores, que contribuem para o encontro de sentidos no mundo social do morar^(10)^.

No entanto, ao pensar o espaço do morar no SRT na perspectiva da desinstitucionalização, há desafios vividos pelos profissionais da equipe multiprofissional, tais como os limites do cuidado no CAPS e no SRT devido ao envelhecimento dos moradores, o que aponta para a necessidade de discussões sobre essa demanda. Esse cenário se agrava diante das fragilidades estruturais da RAPS no Brasil, marcada pela insuficiência de oferta e fragmentação dos serviços, por dificuldades na comunicação entre pontos de cuidado e na efetivação de práticas intersetoriais^(2)^. Embora o modelo de desinstitucionalização represente um avanço fundamental no âmbito da RPB, ainda há obstáculos importantes, como a estigmatização, o subfinanciamento e, por vezes, a reprodução da lógica manicomial em serviços substitutivos^(24)^. Nota-se, como achado deste estudo, que o envelhecimento humano surge como desafio no âmbito dos serviços da RAPS, da efetivação do propósito da desinstitucionalização na RPB e do modelo de cuidado vigente, o que evidencia a preocupação dos participantes de que essa discussão esteja presente na construção cotidiana dos serviços de saúde mental. Em tal contexto, destaca-se a necessidade do fortalecimento das práticas intersetoriais, especialmente daquelas voltadas ao cuidado à pessoa idosa, como estratégia fundamental para efetivação do cuidado integral e da continuidade do processo de desinstitucionalização.

Neste cenário da RAPS, em serviços substitutivos como o SRT, os entrevistados descrevem um desgaste dos profissionais diante das tentativas diárias não concretizadas de construir e implementar um PTS pensado junto com o morador, atribuindo-o a fatores como déficit de funcionários, questões financeiras, demandas de cada morador e múltiplos diagnósticos psicopatológicos, tornando o cuidado complexo. Na atenção à saúde mental, o PTS se mostra como importante estratégia para garantir aspectos como humanização, integralidade e equidade no cenário brasileiro, permitindo descobrir novos caminhos para cuidar^(25)^. Na realidade dos serviços de saúde, o PTS tem sido elaborado em dissonância com o que é preconizado, destacando-se a pouca participação do usuário, a fragmentação do saber multidisciplinar e a dificuldade em compartilhar informações dos casos para discussão ampliada^(25)^. Os achados deste estudo corroboraram a literatura. Assim, recomenda-se a utilização do PTS como ferramenta estruturante do cuidado a partir das singularidades do morador, pensando dentro desse as dificuldades existentes, como déficit de profissionais e aspectos financeiros. Além disso, desenvolver estudos sobre a efetiva utilização do PTS nos SRTs se mostra também como um aspecto importante a ser investigado.

No que tange aos profissionais que atuam na saúde mental, corresponsáveis pela construção diária da desinstitucionalização, há de se considerar os aspectos dos desafios vividos nos serviços de saúde^(26)^. O estresse, o *burnout* e as relações interprofissionais difíceis^(26)^ se mostram como fatores dificultadores do trabalho entre os profissionais de saúde mental, impactando negativamente suas práticas clínicas^(27)^, sendo que esses reivindicam melhores estruturas físicas, mais recursos humanos qualificados antes do ingresso no serviço e materiais para trabalhar^(28)^. Retoma-se que há lacunas na formação em saúde mental, mas, para pensar mudanças na prática profissional, a inovação e o emprego de diferentes ferramentas de cuidado devem ser instigados desde a academia^(29)^. Assim, para pensar um PTS na prática, é necessário que o profissional encontre tempo disponível para os encontros face a face com o morador^(10)^, na perspectiva de constituir a ação do cuidado, além de dispor de estrutura que possibilite o alcance das metas propostas no PTS.

Ao descreverem os desafios do morador idoso, há uma atitude respeitosa dos participantes ao adentrar a casa, entendendo que o SRT não é uma extensão do CAPS. Contudo, destacam o desafio de pensar o SRT, ao mesmo tempo, como o lar de pessoas e um espaço de cuidado, cabendo o questionamento sobre a inserção de práticas típicas de serviços de saúde nesses dispositivos. Em consonância com a legislação regulamentadora dos SRTs, esses dispositivos devem estar vinculados a algum serviço de saúde, enfatizando que, antes de tudo, são espaços de morar^(3)^. Neste ambiente, devido a fatores clínicos inerentes ao envelhecimento dos moradores, surge a necessidade de outros cuidados de saúde, o que parece desafiar o alcance da proposta do SRT^(3)^, demandando serviços com maior suporte e estrutura profissional. Nesse contexto, identificam-se a carência de reflexões nos serviços de saúde sobre o envelhecimento do morador no SRT no que concerne à complexidade dos cuidados requeridos e a sua abordagem no contexto da desinstitucionalização.

Na perspectiva de cuidar do morador em sua residência, traz-se o desafio de articular os cuidados em rede, a partir do reconhecimento e do viver no território, pois há ainda a presença do estigma e da fragmentação do cuidado ao usuário em sofrimento psíquico. O SRT representa a retomada das pessoas em sofrimento psíquico institucionalizadas junto ao território e à perspectiva de construir vivências biográficas em seu mundo-vida a partir de novas relações estabelecidas^(10)^. Entretanto, a fragmentação do cuidado em saúde mental se mostra ainda como desafio no cotidiano, em que a estruturação da RAPS e as ações intersetoriais presentes podem ser caminhos para sustentar, a longo prazo, o projeto do SRT na RPB, considerando o envelhecimento dos moradores e o sucesso na proposta da desinstitucionalização e reabilitação psicossocial^(1,2,24)^.

Existem também intencionalidades apontadas pelos entrevistados no que tange ao cuidado que gostariam realizar, almejando que possa ser não protocolar, singular e que considere outras atividades na ambiência da casa que estimulem a autonomia dos moradores idosos. Pensando no SRT, ao iniciar um resgate da cidadania do morador, é importante que as ações de cuidado em saúde reforcem a configuração de um lar, não de um local de tratamento, com vistas à produção de novas vivências biográficas e ao desenvolvimento progressivo dos moradores em direção a uma maior autonomia^(22)^. Além disso, há, na realidade brasileira, locais em que parte significativa dos moradores de SRTs não frequenta os CAPS de modo regular, além de estarem sem PTS e não serem incluídos como casos de discussão sistemática da equipe^(7)^. Tais apontamentos reforçam a possibilidade de pensar o cuidado ao morador idoso por meio do PTS, porém, para isso, é necessário ter recursos diversos à disposição para realizar essas ações e alcançar as intencionalidades expressas pelos participantes da pesquisa.

Por fim, os profissionais da equipe multiprofissional buscam melhorar suas ações, apreciam o reconhecimento do morador pelo cuidado realizado e procuram aprimorar aspectos do cotidiano do SRT ou mesmo pensar em propostas de atividade terapêutica na casa. Um dos aspectos do cotidiano apontados pelos profissionais como expressão da intencionalidade de suas ações é a alimentação dos moradores. Coadunando as expectativas dos participantes, os aspectos alimentares influenciam a qualidade de vida das pessoas idosas, além do contexto social e de dificuldades de mastigação e deglutição interferirem diretamente no comportamento alimentar^(30)^. Assim, adotar alimentos com textura modificada é uma estratégia que pode melhorar a ingesta, sendo recomendado personalizar a alimentação de cada idoso conforme sua independência e nível de comprometimento oral^(30)^. Portanto, sugere-se a inclusão de tal aspecto citado pelos participantes como anseio de cuidado a ser realizado na construção do PTS com o morador, valorizando seus desejos e buscando a ação longitudinal da equipe multiprofissional.

Também há outros projetos futuros que motivam os profissionais, como o cuidado com o ambiente externo da casa, ao plantar flores ou ao fazer uma horta. Tais ações de cuidado podem ter reflexo na manutenção do SRT como espaço de morar, não o caracterizando com as marcas tradicionais de um serviço de saúde. Para fortalecer essa perspectiva, é importante que os moradores idosos participem ativamente da tomada de decisão sobre os projetos que querem desenvolver, buscando sentido neles, porque, ao serem iniciativas somente da equipe, pode ocorrer resistência do morador em se envolver na atividade como uma forma de defesa de sua autonomia^(22)^.

### Limitações do estudo

Em relação às limitações do estudo, destacamos a abordagem de alguns SRTs de um município, não abrangendo a totalidade dos dispositivos existentes. Identificam-se a demanda de ampliar as investigações sobre a temática abordada, considerando os aspectos de gestão, trabalho e do morar nos SRTs diante da presença de moradores idosos, bem como a necessidade de refletir acerca do envelhecimento junto ao movimento global de desinstitucionalização psiquiátrica.

### Contribuições para a Área

Este estudo evidencia as ações desenvolvidas pela equipe multiprofissional composta, inclusive, pelos profissionais de enfermagem, que diariamente se ocupam de pensar o processo da desinstitucionalização e do cuidado aos moradores idosos dos SRTs. Destaca-se, assim, a importância de qualificar essas práticas como subsídio para efetivação dos princípios da RPB e fortalecimento de novas perspectivas na saúde mental. A enfermagem, por sua proximidade com o cuidado cotidiano e como principal força de trabalho nos serviços da RAPS, desempenha um papel central na promoção da autonomia, no acompanhamento das demandas de saúde e na qualificação das práticas de cuidado, o que fortalece sua atuação na equipe multiprofissional. Como contribuição, o estudo focaliza no recorte específico junto à população idosa desses dispositivos, por haver, no cenário futuro, o aumento dessas pessoas nos espaços de cuidado. Assim, reafirma-se a importância do trabalho em equipe, da ampliação das ações intersetoriais e da utilização do PTS como ferramenta fundamental para orientar o cuidado integral e centrado nas necessidades dos moradores.

## CONSIDERAÇÕES FINAIS

Neste estudo, foi possível construir o tipo vivido a partir das categorias temáticas que evidenciaram as motivações das vivências dos participantes, ao serem permeadas pelo cuidado cotidiano ao morador idoso do SRT em seu mundo-vida e pelos desafios vividos, além de buscarem a manutenção da singularidade biográfica de cada pessoa no espaço coletivo da residência.

Desvelou-se que o cotidiano dos profissionais é transpassado pelo desconhecido em relação ao SRT no seu primeiro contato com a saúde mental e que, ao realizarem os cuidados aos moradores idosos, há sentimentos negativos, como o medo do paciente psiquiátrico. Entretanto, por meio da relação face a face no dia a dia, ocorre o desenvolvimento de vínculos, e a ação se transforma em experiências positivas, tornando o trabalho gratificante. Além disso, os aspectos biográficos e o acervo de conhecimento dos moradores idosos devem ser considerados pelos profissionais no cotidiano, preservando as singularidades em meio ao espaço coletivo do morar.

Entre experiências negativas e positivas, a potência do cuidado dos profissionais com os moradores idosos nos SRTs ocorre por meio do encontro com suas vivências, suas subjetividades e pela vinculação desenvolvida entre eles, além da importância do trabalho da equipe multiprofissional na integralidade do cuidado. Apesar de ainda estarem presentes práticas de saúde fragmentadas e centradas no corpo físico, a atuação conjunta da equipe, orientada pelos princípios da desinstitucionalização e RPB, aponta para a ressignificação dos papéis profissionais e uma mudança de paradigma no cuidado em saúde mental.

Os resultados do estudo revelam também que há dificuldades vividas pela equipe multiprofissional, nas quais o envelhecimento dos moradores no SRT é visto como um desafio diante dos limites para cuidar nos dispositivos da RAPS, levando as equipes ao desgaste e à desmotivação profissional. Nesse contexto, o PTS se mostra como ferramenta estruturante para pensar os desafios e as necessidades individuais dos moradores idosos, bem como a dinâmica da residência. Tem-se como achado também a preocupação em não converter o espaço do morar no SRT em serviço de saúde, mantendo a proposta de dispositivo criado na perspectiva da desinstitucionalização, mas que requer a necessidade de aprofundar as reflexões sobre o cuidado ao morador idoso nesse espaço.

O estímulo à autonomia e a valorização da singularidade do morador idoso do SRT se apresentam como perspectivas que norteiam o cuidado dos profissionais, cujas ações buscam ser melhores a cada dia, em um movimento contínuo de construção do espaço do morar, do SRT como dispositivo da desinstitucionalização e da efetivação dos princípios da RPB.

## Data Availability

Os dados de pesquisa só estão disponíveis mediante solicitação.
